# Histological Evaluation of Pulpal Response and Dentin Bridge Formation After Direct Pulp Capping Using Recombinant Amelogenin and Mineral Trioxide Aggregate (MTA)

**DOI:** 10.7759/cureus.54560

**Published:** 2024-02-20

**Authors:** Laila A Bahammam, Waleed Alsharqawi, Hammam A Bahammam, Maha Mounir

**Affiliations:** 1 Department of Endodontics, King Abdulaziz University, Jeddah, SAU; 2 Department of Pediatric Dentistry, King Abdulaziz University, Jeddah, SAU; 3 Department of Oral Diagnostic Sciences, King Abdulaziz University, Jeddah, SAU; 4 Department of Oral Biology, Future University, Cairo, EGY

**Keywords:** amelogenin, pulpal response, mta, direct pulp capping, dentin bridge

## Abstract

The purpose of the study was to compare and histologically investigate pulpal response and dentin bridge formation after direct pulp capping using recombinant amelogenin and mineral trioxide aggregate (MTA). Recombinant amelogenin protein and MTA were used as pulp capping materials in 120 teeth from eight mongrel dogs. Dogs were sacrificed at two different evaluation times. Regenerative changes were evaluated histologically. At two weeks, in contrast to the MTA group, most of the amelogenin group showed moderately formed hard tissue formation and the pulp tissue was completely filling the entire pulp chamber. These results were statistically significant. At two months, all the samples of the amelogenin group showed complete dentin bridge formation and the pulp chamber was filled entirely with tissue-mimicking the authentic pulp in all the specimens of the amelogenin group. These results were statistically significant. In conclusion, direct pulp capping by recombinant amelogenin protein resulted in significantly better regeneration of the dentin-pulp complex than MTA.

## Introduction

The choice of pulp capping material is a significant element for the success of vital pulp therapy. Mineral trioxide aggregate (MTA) is commonly used as a pulp capping material with great clinical success [1-2]. It presents a greater sealing ability, biocompatibility, and induction of the formation of a dentin bridge through the induction of the differentiation of dental pulp cells into odontoblast-like cells [1-2]. However, it has some limitations including long setting time, difficult handling characteristics, and cytotoxicity during the initial setting [3].

Amelogenin, which is the main enamel matrix protein, had initially been thought to be only expressed by ameloblasts [4-5]. However, recent studies have shown that amelogenin is also expressed endogenously by odontoblasts [6-7]. In carious or traumatized human teeth, amelogenin is distributed in the dentinal tubules under the lesion site and is intensely expressed in the newly differentiated odontoblasts [6]. In an in vitro study, amelogenin is strongly expressed in newly differentiated odontoblast-like cells originating from human dental pulp cells [7]. These data propose that amelogenin may act as a signaling molecule not only during the beginning of the hard matrix development, but also during tooth repair and tissue regeneration [8].

Studies have shown that amelogenin and enamel matrix derivatives (EMD) stimulate odontogenesis through the up-regulation of Osterix and Runx2 [9]. However, their role in endodontic regeneration is still under investigation. Histological studies have shown that amelogenin results in tertiary dentin formation and promotes the induction of odontoblasts and blood capillaries of the dental pulp [10-11]. Amelogenin protein regulates the inflammatory response and enhances the repair capability of odontoblasts [12]. The application of amelogenin protein in endodontics was shown in Mounir et al.’s study [13]. Their results showed that recombinant amelogenin protein enhanced the apex formation of dogs’ immature teeth and promoted soft connective tissue regeneration of the dental pulp [13]. However, the effect of using it as a pulp capping material has not been studied. Therefore, this study aimed to compare and histologically investigate pulpal response and dentin bridge formation after direct pulp capping using recombinant amelogenin and MTA.

## Materials and methods

Ethical approval

Ethical approval was obtained from the Research Ethics Committee (Proposal No. 043 - 2020).

Study sample

Eight 2-year-old mongrel dogs were used in the study. Each dog weighed approximately 25 kg. Split-mouth randomization design was used with a total of 120 teeth.

The tested materials used in the study were ProRoot MTA (Tulsa Dental Products, Tulsa, OK, USA) and recombinant amelogenin protein (obtained from Dr. Malcolm L. Snead, Center for Craniofacial Molecular Biology, Herman Ostrow School of Dentistry of USC, University of Southern California). All the materials were randomly assigned to each quadrant of each dog using a simple randomization technique to allow for a fair distribution of each material.

Animal preparation

Animals were housed under proper veterinarian observation for a week before the experiment to ensure their fitness to the procedure. Prior to the direct-pulp capping procedure, general anesthesia was achieved by intramuscular injection of 10 mL of ketamine hydrochloride (Ketalar; Parke-Davis, Morris Plains, NJ, USA). Each anesthetized dog was placed at the operation site to allow for effective visualization and illumination. Immediately after the procedure, each animal received a Voltaren analgesic (Novartis Pharma, Basel, Switzerland). All the animals were kept on a soft diet to prevent the operative field from being affected by a local injury.

Direct-pulp-capping procedure

All the procedures were performed by one operator. Teeth were isolated with a rubber dam and disinfected with alcohol swabs to ensure a sterile operative field. Standard class V cavities were prepared on the labial surfaces using a sterile fissure bur under water coolant. Pulp exposures of 1 mm diameter were performed with a sterile round carbide bur. Bleeding was controlled by the application of pressure with cotton pellets. The two tested materials (amelogenin and MTA) were prepared according to the manufacturer’s instructions. All the materials were applied equally to the pulp-exposure sites. Pulp exposures of the control group were left empty. The cavities of all groups were sealed by Glass ionomer cement (Fuji 9, GC America, USA). After 14 and 60 days, an overdose of thiopental sodium was administered to the animals for sacrifice after each postoperative period.

Samples preparation

The maxillary and mandibular jaws were dissected, cut into two halves (using a water-cooled diamond disc), and fixed in 10% formalin. The apical foramina and the prepared cavities were carefully covered with wax and teeth were demineralized in Epredia™ TBD-1 decalcifier (Fisher Scientific, Waltham, MA, USA) for 30 minutes according to manufacturer’s instructions. Then, each tooth was sectioned buccolingually. Sections were then stained with hematoxylin and eosin and trichrome stains.

Histological evaluation

Two-week and two-month evaluation periods were selected to evaluate the early and late histological changes in the dentine-pulpal complex. The stained sections were histologically evaluated by two independent evaluators other than the operator who performed the procedure to avoid bias. The evaluation was done under a light microscope using specific criteria for measuring dentin bridge formation, pulp status, and pulpal inflammatory reaction (Tables 1-3) [14].

**Table 1:** Criteria used to evaluate dentin bridge formation

	Criteria for Dentin Bridge Formation
Score 1	Hard tissue deposition as complete and continuous dentin bridge
Score 2	Hard tissue deposition as incomplete and discontinuous dentin bridge
Score 3	A layer of scattered and foggy hard tissue deposition
Score 4	Lack of hard tissue deposition

**Table 2:** Criteria used to evaluate pulpal inflammatory reaction

	Criteria for Pulpal Inflammatory Reaction
Score 0	Absence of inflammatory cells
Score 1	Mild scattering of inflammatory cells with no structural damage
Score 2	Moderate focal accumulations of inflammatory cells, no tissue necrosis with some disruption of the structure
Score 3	Severe extensive inflammatory cell infiltrates or no recognizable inflammatory cells with few pulp residues

**Table 3:** Criteria used to evaluate pulpal status

	Criteria for Pulpal Status
Score 1	Presence of pulp tissue filling the whole pulp chamber
Score 2	Presence of pulp tissue incompletely filling the pulp chamber with the presence of voids or complete pulp necrosis with few fibrous pulp residues
Score 3	Empty pulp space

Statistical analysis

Data were collected and statistically analyzed using the Chi-square test and considered significant when p value ≤0.05. IBM® SPSS® Statistics Version 20 for Windows (IBM Corp., Armonk, NY, USA) was used.

## Results

Dentin bridge formation

Two-Week Observation Period

**Figure 1:**
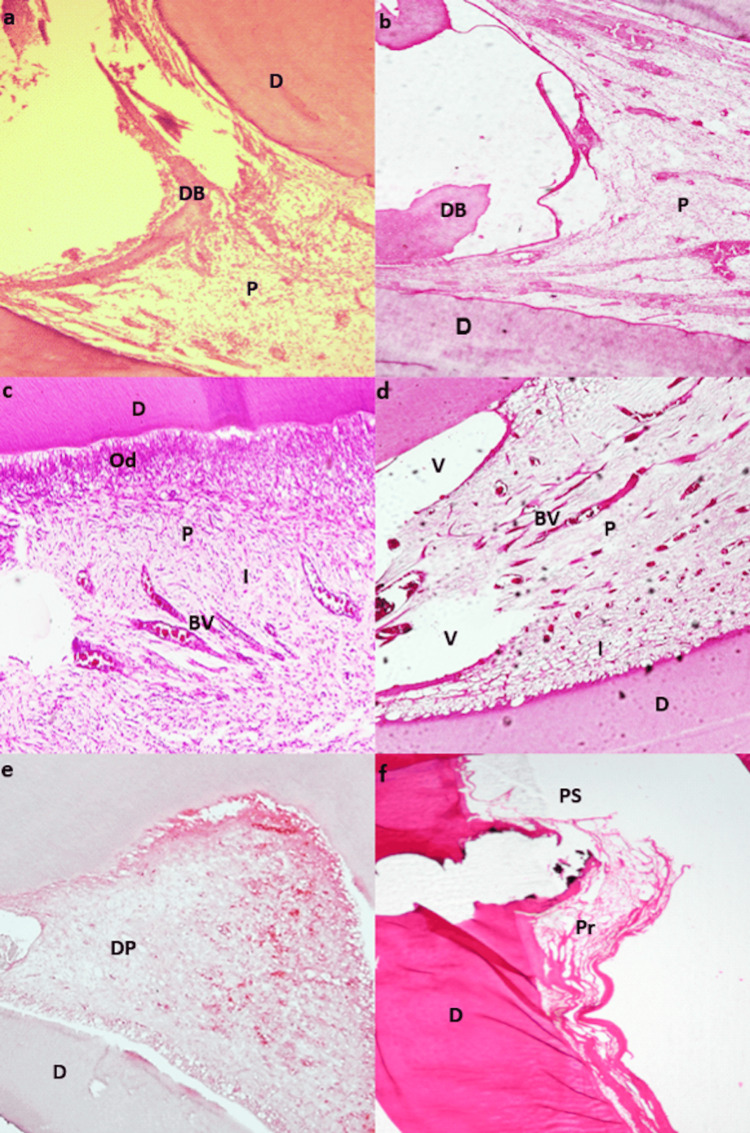
Teeth capped with amelogenin, MTA, and negative control group two weeks post-treatment. Teeth capped with amelogenin two weeks post-treatment. a. Tooth showing nearly complete dentin bridge (DB). Pulp (P) fills the whole pulp chamber. Dentin (D). H&E stain. b. Tooth showing incomplete dentin bridge (DB), pulp (P) and dentin (D). H&E stain. Teeth capped with MTA two weeks post-treatment. c. Tooth showing no dentin bridge formation. Pulp (P) shows dilated engorged blood vessels (BV) and inflammatory cell infiltrate (I). Dentin D. H&E stain. d. Tooth showing no dentin bridge. Coronal pulp (P) shows multiple vacuoles (V). Dentin (D) and inflammatory cell infiltrate (I). H&E stain. Negative control group two weeks post-treatment. e. Tooth showing no dentin bridge formation. Degenerating pulp (DP). Dentin (D). H&E stain. f. Tooth showing no dentin bridge formation. Pulp space (PS) shows pulp residues (Pr). Dentin (D). H&E stain.

Histological evaluation revealed that none of the three groups had complete dentin bridge formation (score 1) at the 2-week observation period. However, most of the amelogenin group (80%) showed moderately formed hard tissue deposition (score 2), and the rest (20%) had slight hard tissue deposition (score 3). On the other hand, the MTA group showed either a slight hard tissue deposition (score 3) (55%) or a lack of hard tissue deposition (score 4) (45%). Whereas, the control group showed a lack of hard tissue deposition (score 4) in all of the cases (100%). These results were statistically significant (P=0.001) (Table 1 & Table 4) (Figure 1).

**Table 4:** Descriptive statistics and Chi-square test results of hard tissue formation of the tested materials. MTA: Mineral trioxide aggregate

Materials Used	Evaluation Periods	Hard Tissue Formation				Total	P-value
		Score 1	Score 2	Score 3	Score 4		
Amelogenin	Two Weeks	0 (0%)	16 (80%)	4 (20%)	0 (0%)	20	0.001
	Two Months	20 (100%)	0 (0%)	0 (0%)	0 (0%)	20	0.001
MTA	Two Weeks	0 (0%)	0 (0%)	11 (55%)	9 (45%)	20	0.001
	Two Months	11 (55%)	8 (40%)	0 (0%)	1 (5%)	20	0.001
Control	Two Weeks	0 (0%)	0 (0%)	0 (0%)	20 (100%)	20	0.001
	Two Months	0 (0%)	0 (0%)	0 (0%)	20 (100%)	20	0.001

Two-Month Observation Period

**Figure 2:**
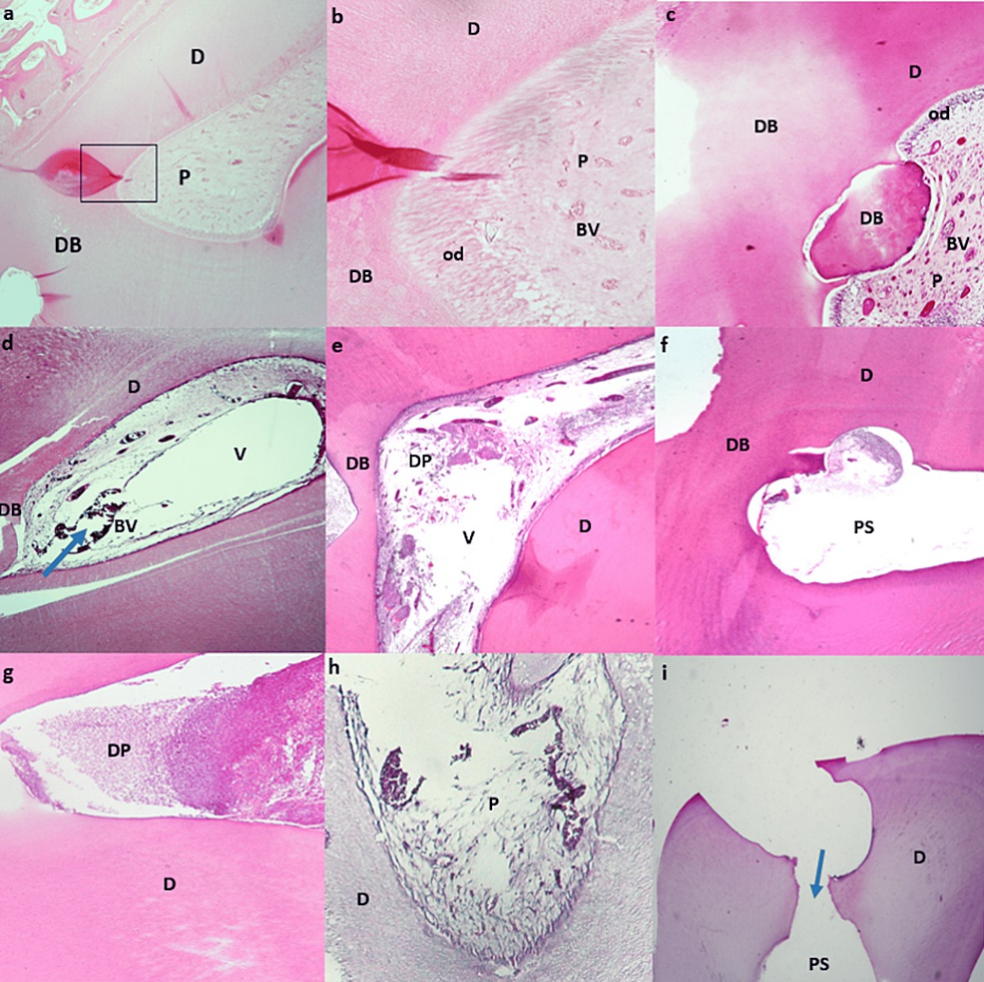
Teeth capped with amelogenin, MTA, and negative control group two months post-treatment. Teeth capped with amelogenin two months post-treatment. a. Tooth showing dentin bridge (DB) beneath the cavity, pulp (P) fills the whole pulp chamber, blood vessels (BV) and dentin (D). H&E stain. b. Higher magnification of the box in panel (a) showing odontoblasts (od) lining the pulp (P) periphery under the dentin bridge (DB) and blood vessels (BV). H&E stain. c. Tooth showing dentin bridges (DB), odontoblasts (od) lining the pulp periphery (P) filling the whole chamber, blood vessels (BV) and dentin (D). H&E stain. Teeth capped with MTA two months post-treatment. d. Tooth showing dentin bridge (DB) beneath the cavity, pulp (P) shows dilated engorged blood vessels (BV) (arrow) and empty vacuoles (V). Dentin (D). H&E stain. e. Tooth showing dentin bridge (DB), degenerating pulp (DP) containing vacuoles (V). Dentin (D). H&E stain. f. Tooth showing dentin bridge (DB), pulp chamber shows space of degenerated pulp tissue (PS). Dentin (D). H&E stain. Negative control group two months post-treatment. g. Tooth showing no dentin bridge, pulp chamber is filled with degenerating pulp tissue (DP), dentin (D). H&E stain. h. Tooth showing no dentin bridge formation, pulp (P) shows signs of degeneration and vacuole formation. Dentin (D). H&E stain. i. Tooth showing no dentin bridge formation (arrow), pulp is devoid of pulp tissue showing an empty pulp space (PS) and dentin (D). H&E stain.

Histological evaluation revealed that all the samples of the amelogenin group (100%) showed complete dentin bridge deposition (score 1). However, most of the specimens of the MTA group (55%) showed complete dentin bridge deposition (score 1). In contrast, 40% of the MTA samples had moderately formed hard tissue deposition (score 2). One sample of the MTA group did not show any hard tissue deposition (score 4). Whereas, the control group showed a lack of hard tissue deposition (score 4) in all of the cases (100%). These results were statistically significant (P=0.001) (Table 1 & Table 4) (Figure 2).

Inflammatory reaction

Two-Week Observation Period

Histological evaluation of the inflammatory reaction revealed that none of the specimens in all groups had an absence of inflammatory cells (score 0) after two weeks. Mild scattering of inflammatory cells with no structural damage (score 1) was seen in all the specimens in the amelogenin group. Moderate focal accumulations of inflammatory cells with some disruption of the structure without tissue necrosis (score 2) were seen in the majority of MTA specimens (75%). The remaining 25% of the MTA group had a mild scattering of inflammatory cells with no structural damage (score 1). However, the control group showed mild to moderate scattering of inflammatory cells accompanied by structural damage (score 2) in 100% of the cases. These results were statistically significant (P=0.001) (Table 2 & Table 5) (Figure 1).

**Table 5:** Descriptive statistics and Chi-square test results of inflammatory reaction of the tested materials.

Materials Used	Evaluation Periods	Inflammatory Reaction				Total	P-value
		Score 0	Score 1	Score 2	Score 3		
Amelogenin	Two Weeks	0 (0%)	20 (100%)	0 (0%)	0 (0%)	20	0.001
	Two Months	20 (100%)	0 (0%)	0 (0%)	0 (0%)	20	0.98
MTA	Two Weeks	0 (0%)	5 (25%)	15 (75%)	0 (0%)	20	0.001
	Two Months	19 (95%)	1 (5%)	0 (0%)	0 (0%)	20	0.98
Control	Two Weeks	0 (0%)	0 (0%)	20 (100%)	0 (0%)	20	0.001
	Two Months	0 (0%)	0 (0%)	0 (0%)	0 (0%)	20	0.98

Two-Month Observation Period

After two months, the inflammatory reaction decreased in amelogenin and MTA groups. Almost all of the samples in both groups had an absence of inflammatory cells (score 0). The differences between both groups were not statistically significant (P=0.98). One sample from the MTA group had a mild scattering of inflammatory cells with no structural damage (score 1). On the other hand, the control group showed few pulp residues with no recognizable inflammatory cells (score 3) in 100% of the cases (Table 2 & Table 5) (Figure 2).

Pulp status

Two-Week Observation Period

Several histological features were observed within the pulp after two weeks. An odontoblastic layer was seen in all the specimens of amelogenin and MTA groups. The presence of calcification was found mainly in the amelogenin group and some of the MTA group. Moreover, dilated blood vessels were frequently seen in both groups with the presence of inflammatory cells.

Pulp tissue was found completely filling the entire pulp chamber (score 1) in all the specimens of the amelogenin group (100%), and amelogenin was seen in direct contact with the pulp tissue in all the samples. However, most of the specimens in the MTA group (75%) showed incomplete filling of the whole pulp along with empty pulpal spaces (score 2), and there was always a space between the MTA material and the pulp tissue. The control group showed nearly complete pulp necrosis with few fibrous pulp residues (score 2) in 100% of the cases. These results were statistically significant (P=0.001) (Table 3 & Table 6) (Figure 1).

Two-Month Observation Period

After a two-month observation period, the histological findings indicated the presence of odontoblast-like cells in most of the amelogenin specimens. Large dilated blood vessels were observed in the MTA group. The pulp chamber was completely filled with tissue mimicking the authentic pulp (score 1) in all the specimens of the amelogenin group (100%). Amelogenin was observed in direct contact with the pulp tissue in all the specimens. On the other hand, 60% of the specimens in the MTA group had pulp tissue incompletely filling the pulp chamber with the presence of voids (score 2), and the remaining 40% had almost completely empty pulpal spaces (score 3), indicating complete loss of pulp tissue regardless of the presence of dentinal bridging. The architecture of the pulp was not preserved in most of the MTA group, and the regenerated pulp did not resemble the authentic pulp. On the other hand, 80% of the specimens in the control group had almost completely empty pulpal spaces (score 3) and 20% had tissue that incompletely filled the pulp chamber which did not resemble the authentic pulp with the presence of voids (score 2). These results were statistically significant (P=0.001) (Table 3 & Table 6) (Figure 2).

**Table 6:** Descriptive statistics and Chi-square test results of pulp status of the tested materials.

Materials Used	Evaluation Periods	Pulp Status			Total	P-value
		Score 1	Score 2	Score 3		
Amelogenin	Two Weeks	20 (100%)	0 (0%)	0 (0%)	20	0.001
	Two Months	20 (100%)	0 (0%)	0 (0%)	20	0.001
MTA	Two Weeks	5 (25%)	15 (75%)	0 (0%)	20	0.001
	Two Months	0 (0%)	12 (60%)	8 (40%)	20	0.001
Control	Two Weeks	0 (0%)	20 (100%)	0 (0%)	20	0.001
	Two Months	0 (0%)	4 (20%)	16 (80%)	20	0.001

## Discussion

Researchers always seek new treatment modalities that may improve treatment outcomes. They improvise new techniques, use new materials, and investigate the capabilities of the dental tissues to restore the original architecture and biological function of the injured tissues or organs based on cellular and molecular biology. A novel approach is proposed using recombinant amelogenin protein as a pulp capping material based on its bio-activity in regulating intracellular signal pathways, resulting in a chain of molecular signaling events that ultimately alter target proteins not only during the initiation of hard matrix formation, but also during tooth repair and tissue regeneration [8-12]. Moreover, amelogenins can reduce the levels of pro-inflammatory cytokines [15] and increase the synthesis of growth factors such as growth factor-β1 [16] and vascular endothelial growth factor [17] which are the key components of the healing response.

This animal study was conducted to evaluate the regenerative capacity of recombinant amelogenin protein when used as a direct pulp-capping agent in comparison to a commonly used capping material, MTA. We histologically investigated dentin bridge formation, inflammatory reaction, and pulpal response after using the tested materials. Based on our observations, the pulp status criterion was added to the methodology. Because dentin bridging was a common feature in the MTA group the pulp under the MTA, when present, did not resemble the authentic pulp in most of the cases.

Two evaluation periods were selected in our study to evaluate the early and delayed effects of the capping materials on the pulp. Several methods have been previously used to evaluate the success of direct pulp capping. These methods include radiographs [18], micro-CT [2], and histological evaluation [1-2,19]. Histological evaluation was the evaluation method used in this study because it is more reliable than radiographs in the detection of dentin bridging [19] and provides more information about the regenerative features following the application of each tested material.

The recombinant amelogenin protein group showed significantly better dentin bridging than the MTA group after both observation periods. Interestingly, complete dentin bridge formation was significantly evident in all the samples of the amelogenin protein group after two months. Previous studies reported that amelogenin can act as a molecule for guiding the cells to play a role in the repair or regeneration of dentin and cementum [20-22]. Others described its success in periodontal regeneration [22]. Amelogenin isoform is one of the basic components of EMD. Two studies [10-11] reported favorable histological outcomes following the application of EMD as a direct pulp capping material in animals. Al-Hezaimi et al. [23] reported better outcomes after combining EMD with MTA in a direct pulp capping procedure compared to using MTA alone. Micro-CT and histological evaluation were used in their study, and their results showed a lack of tunnel defects after both materials were combined.

Mounir et al. [13] evaluated the effect of recombinant amelogenin in pulpal and apical regeneration of immature necrotic teeth of young mongrel dogs. Their findings indicated that recombinant amelogenin promoted connective tissue regeneration and apical closure. In our study, the predominant picture of the first observation period for the recombinant amelogenin group was a mild scattering of inflammatory cells and angiogenesis, with no structural damage (score 1). The material was in direct contact with the pulp tissue. Mild inflammation disappeared in the second observation period. An authentic architecture of the pulp was observed, completely filling the pulp chamber with dentin-like cells lining the dentin bridge.

MTA has been reported to be the gold standard in the direct pulp capping procedure [1]. Several studies have reported its histological and clinical success as a direct pulp capping material [14,24]. In our study, MTA resulted in a slightly formed dentin bridge at the first observation period, and after two months, hard tissue barrier formation increased significantly. All of the samples had either complete or moderately formed dentin bridge formation. Histological findings of previous studies indicate better dentin bridge formation after a long follow-up period, which is in agreement with our findings [1,14,25]. Koh et al. [26] reported that the hard tissue formation of MTA is strongly related to the properties of MTA, such as biocompatibility, alkalinity, and sealing ability, which prevent bacterial microleakage and pulpal inflammation [27]. Min et al. [28] found that induction of hard tissue after the application of MTA is related to Portland cement, which is considered as one of the main MTA components.

In our study, most of the specimens of the MTA group showed moderate focal accumulation of inflammatory cells with tissue necrosis and some disruption of the structure of the pulp. Most of the MTA samples had voids and there was always a space between the MTA material and the pulp tissue. Forty percent of the samples in the second observation period had nearly empty pulp spaces, indicating a complete loss of pulp tissue regardless of the presence of dentinal bridging. This may be attributed to the fact that the material leaches toxic metals in the nearby environment [29], which leads to tissue damage over time.

Most of the specimens in the recombinant amelogenin and MTA groups were observed to have dilated blood vessels at the two-week observation period. This could be interpreted as an early inflammatory tissue reaction to both materials or as a sequel to the operative procedures and placement of both materials. On the other hand, during the two-month observation period, dilated blood vessels were noted to be significant in the MTA group only. This could be interpreted as pulp hyperemia, which is the first sign of pulp degeneration. Dentinal bridging may be indicative of a healing process or sign of irritation [24]. These findings may be attributed to the increased alkalinity of the material or the exothermic reaction of the MTA, which leads to an extreme increase in the pulp temperature that results in ischemia and necrosis, coupled with the harmful effects of MTA [30]. On the other hand, the control group showed a poor prognosis; a lack of hard tissue deposition, and the underlying pulp tissue showed necrosis with few fibrous pulp residues.

Previous studies used radiographs [18], Micro-CT [2], and histological evaluation [1-2,19] to test dentin bridge formation. In this study, we used histological evaluation because it is more reliable than radiographs in detecting dentin bridging [19] and provides more comprehensive information about the regenerative features following the application of each tested material. It is the only way to accurately assess the pulpal response toward both materials.

In most previous studies, the criteria for the healing of the exposed pulp are normally included, i.e., the formation of a hard tissue barrier covering the wound area [25] and the absence of inflammation in the subjacent tissue [1,14,25] regardless of the condition of the pulp underneath the capping material. The ideal capping material should preserve the remaining pulp and induce healing of the wounded pulp in addition to forming a dentin bridge. Although our findings were obtained from histological evaluation, which is considered a reliable method for evaluation, we should respect the fact that efforts should be directed to incorporate bacteria into the previous model to prove the efficacy of the material.

Limitation

Although our findings were obtained from histological evaluation, which is considered a reliable evaluation method, the limitation of the study was that we did not respect the fact that efforts should be directed to perform this study in humans and incorporate bacteria into the previous model to prove the efficacy of the material. However, we did not deliberately set in infection as the outcome with MTA was questionable in this case, and the effect of amelogenin was tested in a previous study using infected root canals, and the results were favorable [13].

## Conclusions

The success of direct pulp capping is based on dentin bridge formation and preservation of the authentic pulp architecture as a result of an insult to the pulp tissue due to exposure. The ability of the pulp to regenerate under the exposure site and form a reparative dentin bridge is a sign of complete healing and regeneration. In both test time groups, the amelogenin fulfilled these criteria. In the MTA group, although a dentin bridge was formed, the pulp tissue was not restored to its original pulp architecture. It may be concluded from this study that direct pulp capping using amelogenin resulted in significantly better results leading to the regeneration of the dentin-pulp complex compared to MTA, thus restoring the biological functions of the affected tooth.
